# Transcription Factors Fzc9 and Pdr802 Regulate ATP Levels and Metabolism in *Cryptococcus neoformans*

**DOI:** 10.3390/ijms26051824

**Published:** 2025-02-20

**Authors:** Yuanyuan Ma, Peng Xue

**Affiliations:** Nantong Key Laboratory of Environmental Toxicology, Department of Occupational Medicine and Environmental Toxicology, School of Public Health, Nantong University, Nantong 226019, China

**Keywords:** *Cryptococcus neoformans*, transcription factor, Fzc9, Pdr802, metabolism, ATP

## Abstract

Transcription factors Fzc9 and Pdr802, characterized by their Zn_2_Cys_6_ DNA-binding domain, are essential for the virulence of *Cryptococcus neoformans* in lung and brain infections. Notably, the in vivo roles of Fzc9 and Pdr802 in contributing to the pathogenicity of *C. neoformans* are not adequately reflected by the phenotypic characteristics observed in vitro. This study investigates the effects of gene deletion of *FZC9* or *PDR802* on the proteomic and metabolomic profiles of *C. neoformans*. Using mass spectrometry analysis, we identified significant changes in protein abundance and metabolite levels, particularly in pathways related to ATP synthesis. These findings deepen our understanding of the metabolic roles of Fzc9 and Pdr802, suggesting potential targets for the development of novel therapeutic strategies against *C. neoformans* infections.

## 1. Introduction

Invasive fungal infections are a significant yet often overlooked public health threat. Recent estimates indicate that there are approximately 6.5 million cases of these infections each year, leading to around 3.8 million deaths worldwide. Alarmingly, about 2.5 million of these deaths—representing 68% of the total, with a range from 35% to 90%—can be directly attributed to these infections [1,2]. Among these pathogens, *Cryptococcus neoformans* stands out as a major opportunistic fungal pathogen, accounting for more than 220,000 infections and 180,000 deaths worldwide each year, particularly affecting individuals with HIV/AIDS [3,4,5]. Recognized by the World Health Organization as one of the four critical priority fungal pathogens [6], *C. neoformans* initiates infection upon inhalation, leading to colonization of the lungs and subsequent dissemination to the brain [7]. The pathogenicity is significantly influenced by its unique cell surface characteristics. Notable among these are the deposition of melanin in the cell wall and the attachment of polysaccharides in the capsule layer, both of which contribute to its virulence by helping the organism evade the host immune response [8,9]. Despite the availability of antifungal treatments, managing cryptococcosis remains challenging due to a limited number of effective drugs, the emergence of drug resistance, and the absence of effective vaccines [10].

*C. neoformans* utilizes complex signaling networks to cross the blood-brain barrier (BBB) and establish infection. Nine transcription factors (TFs) have been identified as core virulence kinases essential for infection in both the lungs and brain [11]. Key TFs include Hxl1 in the unfolded protein response pathway [12], Hob1 and Sre1 in the sterol biosynthesis pathway [13], as well as Gat201 and Nrg1 in the capsule biosynthesis pathway [14,15]. Alongside the well-characterized TFs, Fzc9 and Pdr802, which are both characterized by the fungal-specific Zn_2_Cys_6_ DNA-binding domain, have also been identified as crucial virulence factors. Although the in vivo functions of Fzc9 and Pdr802 in the pathogenicity remain inadequately understood, primarily due to the limitations of in vitro studies on mutant phenotypes, both factors have proven critical for traversing the BBB, a crucial step for fungal pathogenicity [11,13]. Notably, the deletion of *FZC9* impairs mating ability and increases sensitivity to hydrogen peroxide, suggesting its essential role in both reproductive and stress response mechanisms [11]. In this study, we investigate the roles of the TFs Fzc9 and Pdr802 in the *C. neoformans* through proteomic and metabolomic analyses. We employed data-independent acquisition (DIA), a robust method for quantitative proteomics that allows for comprehensive protein profiling, alongside untargeted metabolomics to compare WT strains with *fzc9*Δ and *pdr802*Δ mutants. We found significant alterations in protein expression and metabolite profiles, emphasizing the essential roles of Fzc9 and Pdr802 in regulating cellular metabolism and virulence. Specifically, the loss of Fzc9 and Pdr802 leads to substantial changes in the expression of key proteins and metabolites involved in metabolic pathways, including oxidative phosphorylation, purine metabolism, and the tricarboxylic acid (TCA) cycle. This disruption results in a marked decrease in intracellular ATP levels. These results not only elucidate the complex relationship between these TFs and mitochondrial function but also highlight potential therapeutic interventions, such as metabolic inhibitors or modulators, targeting metabolic processes in *C. neoformans*.

## 2. Results

### 2.1. Proteomic and Metabolomic Investigations of the fzc9Δ Mutant

**Figure 1:**
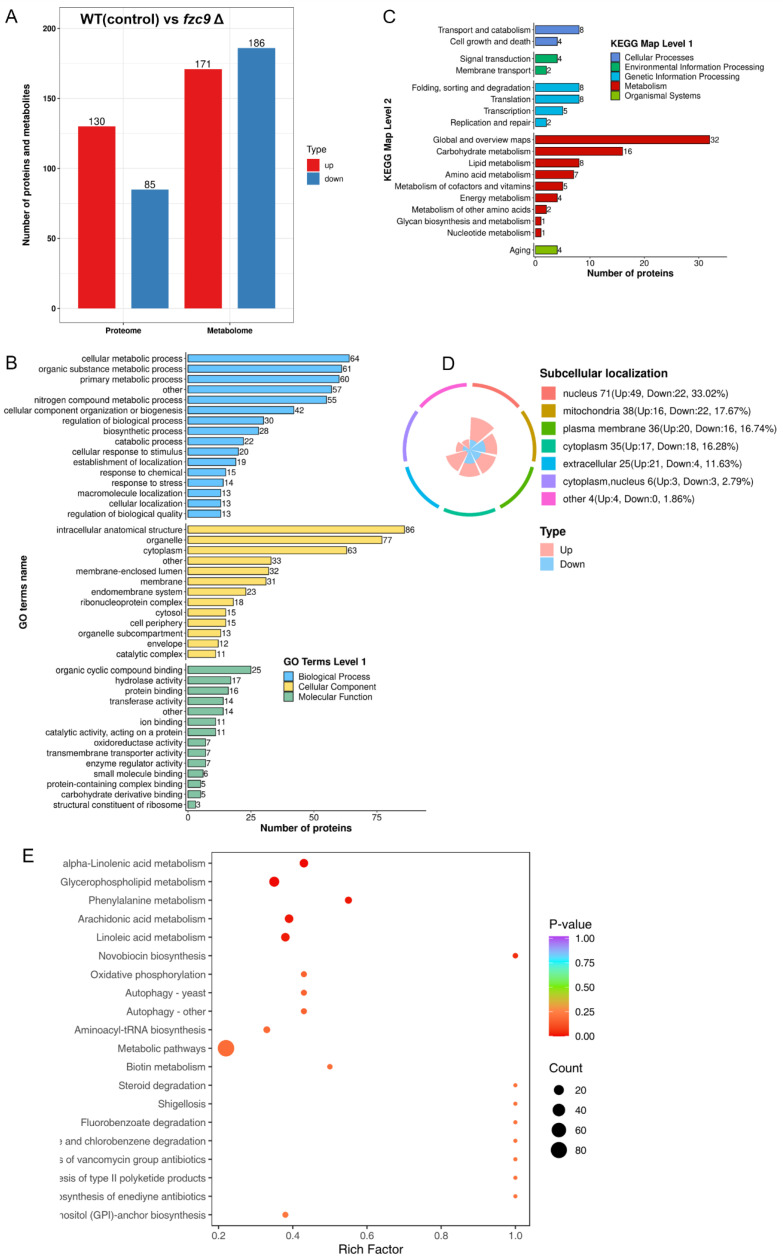
The knockout of the *FZC9* gene led to significant alterations in the protein and metabolic profiles in *C. neoformans*. The bar chart illustrates the number of differentially expressed proteins and metabolites. For the proteomic analysis, proteins were classified as significantly up-regulated (indicated in red) if the ratio of the experimental group to the control group exceeded 1.5 with t-test probabilities of less than 0.05. Conversely, proteins were deemed significantly down-regulated (indicated in blue) if the ratio was less than 0.67 with t-test probabilities also below 0.05. In the metabolomic analysis, metabolites were classified similarly, with up-regulated metabolites shown in red and down-regulated metabolites in blue, based on t-test probabilities of less than 0.05 (**A**). GO classification. The GO classification of regulated proteins illustrates a functional enrichment analysis, highlighting the biological processes, molecular functions, and cellular components associated with differentially expressed proteins (**B**). KEGG classification. This analysis further indicates the involvement of regulated proteins in specific processes, providing insights into functional enrichment (**C**). Subcellular localization. The subcellular localization of differentially expressed proteins sheds light on where these proteins are active within the cell, offering context for their functional roles (**D**). KEGG enrichment analysis of metabolites. The KEGG enrichment analysis of differentially expressed metabolites underscores the metabolic pathways affected by the *FZC9* gene knockout (**E**).

The TF Fzc9 is crucial for the pathogenicity of *C. neoformans* in lung and brain infections [11]. Interestingly, deletion of the *FZC9* gene does not produce significant phenotypic changes pertaining to virulence. This study aimed to elucidate the effects of *FZC9* deletion on the proteomic and metabolomic profiles of *C. neoformans*. Using DIA for quantitative proteomics, we conducted a comparative analysis of WT strain and *fzc9*Δ mutants. This approach allowed us to identify proteins exhibiting differential expression, revealing a total of 4931 proteins from the 7429 proteins annotated in *C. neoformans*. We defined significant changes in protein expression with thresholds for expression ratios below 0.67 or above 1.5, along with a significance criterion of *p* < 0.05 based on t-test results. A summary of the identified differences in protein expression is provided in Table S1. Notably, the absence of *FZC9* led to alterations in the expression of proteins linked to 215 genes, including 130 upregulated and 85 downregulated proteins (Figure 1A). GO analysis indicated that these differentially expressed proteins are involved in various biological processes, such as cellular metabolic processes, primary metabolic functions, organic metabolism, and nitrogen compound metabolism. Additionally, these proteins were categorized by their cellular components and molecular functions (Figure 1B). KEGG pathway analysis revealed that the altered proteins are associated with multiple metabolic pathways, including carbohydrate metabolism, energy metabolism, lipid metabolism, amino acid metabolism, glycan biosynthesis, cofactor and vitamin metabolism, and nucleotide metabolism (Figure 1C). We present the distribution of these differentially expressed proteins across subcellular compartments—including the nucleus, mitochondria, plasma membrane, cytoplasm, and extracellular region—in Figure 1D. Turning to the metabolomics component, the deletion of *FZC9* revealed unique metabolic profiles characterized by 357 individual changes: 171 metabolites exhibited elevated levels while 186 showed reduced levels (summarized in Figure 1A). The KEGG enrichment analysis for the differentially expressed metabolites is illustrated in Figure 1E. In summary, the knockout of *FZC9* significantly impacts both protein expression and metabolite profiles in *C. neoformans*, underscoring its potential role in regulating cellular metabolism and virulence.

### 2.2. Proteomic and Metabolomic Investigations of the pdr802Δ Mutant

**Figure 2:**
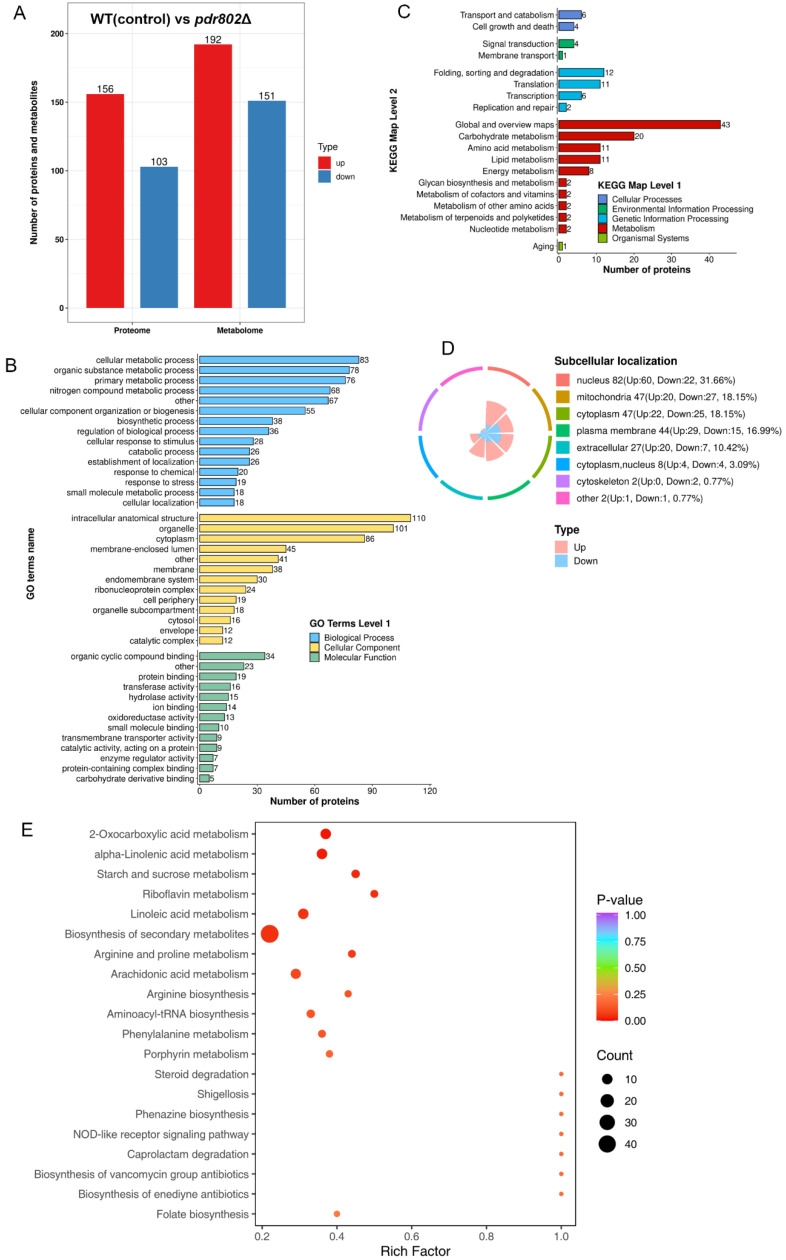
Knockout of the *PDR802* altered protein and metabolite profiles in *C. neoformans*. Differentially expressed proteins and metabolites—up-regulated proteins in red (ratio > 1.5, *p* < 0.05) and down-regulated proteins in blue (ratio < 0.67, *p* < 0.05). Metabolites are shown with *p* < 0.05 (**A**). GO classification. The functional enrichment analysis of differentially expressed proteins (**B**). KEGG enrichment analysis of differentially expressed metabolites in specific processes (**C**). Subcellular localization. The localization of differentially expressed proteins within the cell (**D**). KEGG enrichment analysis of differentially expressed metabolites in specific pathways (**E**).

The TF Pdr802, similar to Fzc9, is involved in key signaling pathways linked to virulence [11]. To explore the regulatory roles of these TFs, we conducted extensive proteomic and metabolomic analyses on the *pdr802*Δ mutant. Our proteomic investigation identified 4931 proteins out of a total of 7429 proteins present in *C. neoformans*. We determined significant changes in protein expression using thresholds of ratios falling below 0.67 or exceeding 1.5, with a significance level of *p* < 0.05 based on t-test probabilities. A summary of these observations is presented in Table S1. The deletion of *PDR802* resulted in the differential expression of proteins in 259 genes, which included 156 upregulated proteins and 103 downregulated proteins (Figure 2A). GO analysis highlighted the involvement of these proteins in various biological processes, including cellular metabolic processes, organic substance metabolism, primary metabolic activities, small molecule metabolic processes and nitrogen compound metabolism. Additionally, proteins were categorized based on their cellular components and molecular functions, detailed in Figure 2B. The KEGG pathway analysis indicated that these regulated proteins are linked to numerous metabolic pathways, such as energy metabolism, carbohydrate metabolism, amino acid metabolism, cofactor and vitamin metabolism, lipid metabolism, nucleotide metabolism, glycan biosynthesis, and terpenoid and polyketide metabolism (Figure 2C). The subcellular localization of the differentially expressed proteins—including those within the nucleus, mitochondria, cytoplasm, plasma membranes, and cytoskeleton—is depicted in Figure 2D. From a metabolomic perspective, the deletion of *PDR802* resulted in distinct metabolic profiles characterized by 343 individual changes, with 192 metabolites showing increased levels and 151 metabolites demonstrating decreased levels (Figure 2A). The KEGG enrichment analysis for these differentially expressed metabolites is presented in Figure 2E. Overall, the knockout of *PDR802* has a profound impact on both protein expression and metabolite levels in *C. neoformans*, underscoring its essential role in regulating cellular metabolism and virulence.

### 2.3. Fzc9 and Pdr802 Regulating Intracellular ATP Levels

**Figure 3:**
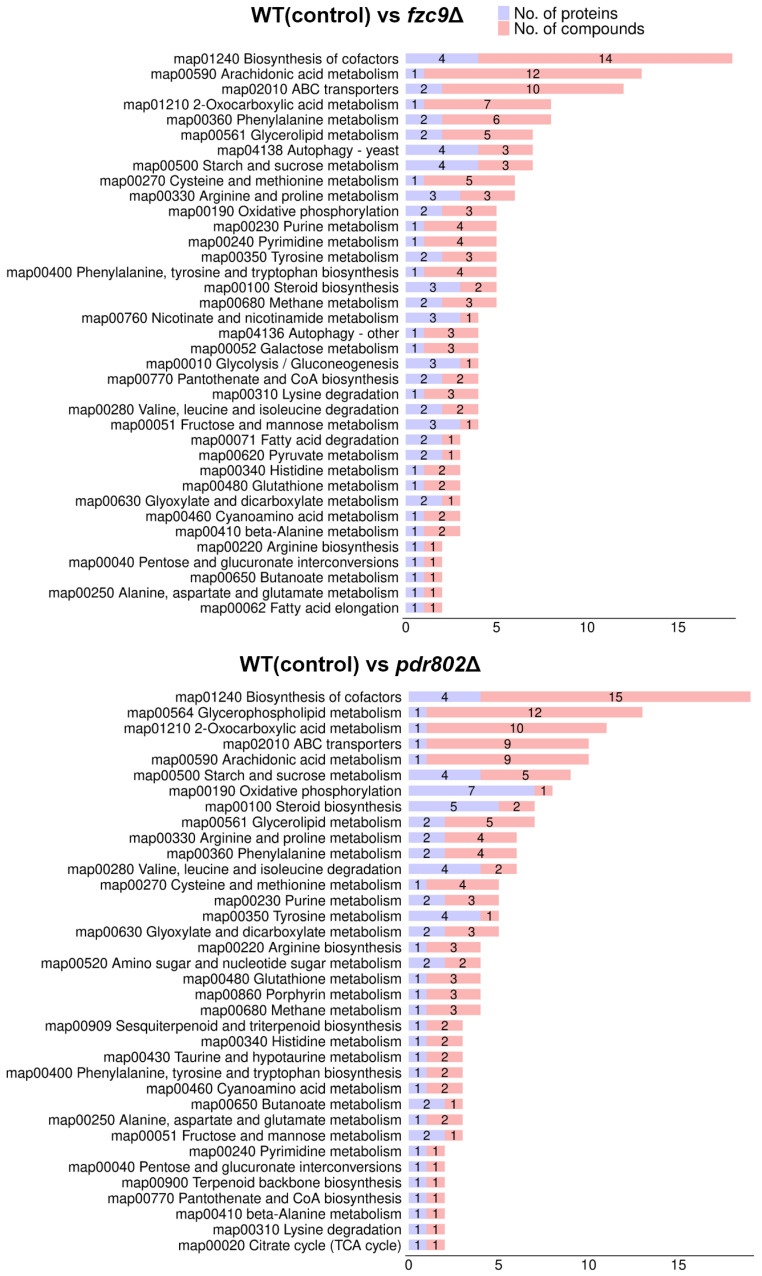
Examination of the relationships between proteomic and metabolomic profiles, categorizing differentially expressed metabolites and proteins by their associated biological pathways.

**Figure 4:**
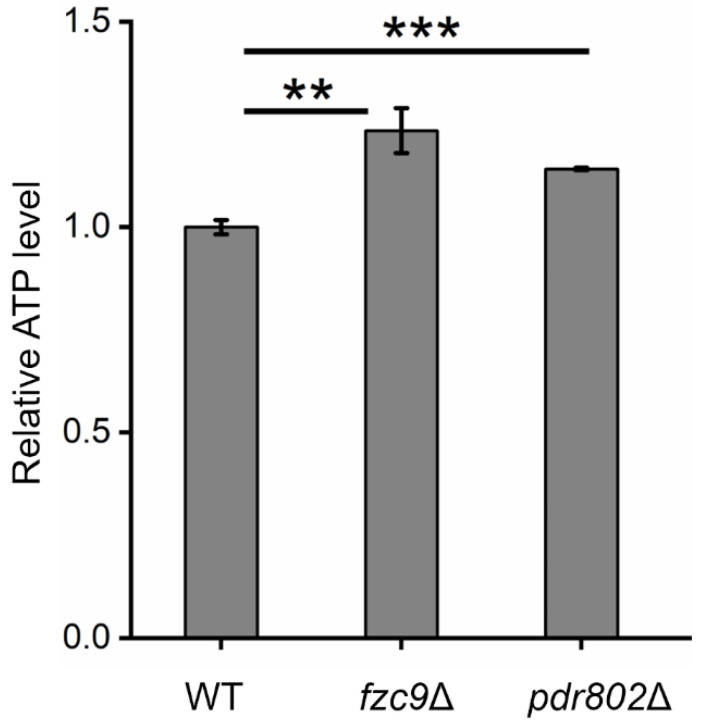
The knockout of *FZC9* or *PDR802* resulted in significant changes in intracellular ATP levels. Significance levels are marked as ** (*p* < 0.01), *** (*p* < 0.001).

Fzc9 and Pdr802 are essential regulators of intracellular ATP levels, as demonstrated by our proteomic and metabolomic analyses. These studies reveal that both transcription factors significantly influence the expression of mitochondrial proteins and the levels of related metabolites. Figure 1D and Figure 2D, along with Tables S2 and S3, illustrate the subcellular localization of differentially expressed proteins, particularly those associated with mitochondria. Notably, Figure 3 highlights mitochondrial proteins and metabolites that are differentially expressed and involved in critical cellular processes, including oxidative phosphorylation, purine metabolism, and the TCA cycle. Mitochondria are recognized as the primary sites for ATP synthesis within the cell. The generation of mitochondrial ATP is closely linked to oxidative phosphorylation, purine metabolism, and the TCA cycle. Oxidative phosphorylation is the key process for ATP production, while the TCA cycle provides the reducing equivalents (NADH and FADH_2_). Purine metabolism contributes to the synthesis of ATP for cellular functions. These three components are interconnected and work synergistically in cellular energy metabolism. Our experiments demonstrated a significant increase in ATP levels following the knockout of either *FZC9* or *PDR802*, as shown in Figure 4. This elevation in ATP levels suggests a nuanced interplay between these transcription factors and mitochondrial function. Specifically, the disruption of *FZC9* or *PDR802* appears to influence mitochondrial protein expression, which in turn boosts ATP production. These findings underscore the critical roles of Fzc9 and Pdr802 in ATP regulation, with broader implications for cellular energy dynamics and virulence. Understanding this relationship may provide insights into potential therapeutic strategies aimed at targeting energy metabolism in pathogenic organisms.

## 3. Discussion

Our study revealed that the TFs Fzc9 and Pdr802 are critical for the pathogenicity of *C. neoformans* by regulating cellular metabolism. Deletion of *FZC9* or *PDR802* leads to significant alterations in protein expression and metabolite profiles, particularly affecting metabolic pathways such as oxidative phosphorylation, purine metabolism, and the TCA cycle. Notably, knocking out either *FZC9* or *PDR802* results in a marked increase in intracellular ATP levels.

Mitochondrial function is vital for fungi to cause disease, particularly in their adaptation to challenges in the mammalian environment, including nutrient limitation, stress responses, and immune evasion [16,17,18,19,20]. *C. neoformans* exemplifies how mitochondrial function impacts fungal pathogenicity [16]. Our results are consistent with previous studies highlighting the importance of mitochondrial roles in fungal pathogenesis. Mitochondria are essential for fungal pathogens to respond to and adapt to stress conditions encountered in mammalian hosts, such as oxygen and nutrient limitations, elevated temperatures, pH variability, and various oxidative and nitrosative stresses [16]. The observed increase in intracellular ATP levels in the *fzc9*Δ and *pdr802*Δ mutants suggests that Fzc9 and Pdr802 regulate mitochondrial energy production. This finding aligns with past reports indicating that mitochondrial ATP synthesis is crucial for the survival and virulence of *C. neoformans*. For example, disruptions in mitochondrial function can hinder the ability of *C. neoformans* to survive in nutrient-poor environments and resist oxidative stress [19]. The regulation of proteins and metabolites associated with oxidative phosphorylation and the TCA cycle supports this notion. However, it is important to emphasize that the balance of mitochondrial ATP synthesis is essential for the optimal functioning of *C. neoformans*. Both excess and insufficient ATP levels can adversely affect pathogen viability and virulence. An overabundance of ATP may lead to metabolic overload, potentially damaging cells and reducing the pathogen adaptability to environmental stresses. Conversely, decreased ATP levels can compromise the energy supply necessary for crucial cellular processes, impairing survival and virulence. Therefore, the precise regulation of ATP synthesis by Fzc9 and Pdr802 is likely critical for maintaining the metabolic homeostasis required for *C. neoformans* to thrive in host environments. Moreover, the differential expression of proteins and metabolites across various metabolic pathways, including carbohydrate, lipid, and amino acid metabolism, highlights the multifaceted roles of Fzc9 and Pdr802 in cellular metabolism. These changes may contribute to the adaptive responses of *C. neoformans* to environmental stressors and its establishment of infection in the host. For instance, upregulation of proteins involved in lipid metabolism may facilitate the utilization of host lipids, while alterations in amino acid metabolism could support the synthesis of essential proteins required for pathogenicity.

The differential expression of proteins and metabolites across various metabolic pathways plays a crucial role in fungal virulence. In particular, the involvement of Fzc9 and Pdr802 in regulating steroid biosynthesis is significant. The regulation of the pathway can influence the integrity and stability of the fungal membrane, which is essential for maintaining cellular function and pathogenicity [21,22,23]. Disruptions in steroid biosynthesis pathways may compromise the structural integrity of the fungal membrane, potentially leading to a reduction in the virulence of *C. neoformans* [23]. Pharmacological modulation of these pathways represents a promising strategy for mitigating fungal virulence. By specifically targeting Fzc9 and Pdr802, we can explore the development of novel antifungal agents that inhibit oxidative phosphorylation and disrupt steroid biosynthesis. Such agents could impair the ability of *C. neoformans* to establish infections and enhance the efficacy of existing treatments. In conclusion, our study underscores the significance of Fzc9 and Pdr802 in regulating mitochondrial function and cellular metabolism in *C. neoformans*. The substantial alterations in protein and metabolite profiles, particularly the increase in intracellular ATP levels, provide valuable insights into the molecular mechanisms underlying the pathogenicity of this fungal pathogen. These findings enhance our understanding of the complex interplay between transcription factors and metabolic pathways while also indicating potential targets for developing novel therapeutic strategies against *C. neoformans* infections. Maintaining the delicate balance of mitochondrial ATP synthesis is crucial for the optimal functioning and pathogenicity of *C. neoformans*, emphasizing the potential therapeutic value of targeting these TFs.

## 4. Materials and Methods

### 4.1. Construction of Strains

We employed the wild-type (WT) strain *Cryptococcus neoformans* var. *grubii* H99, alongside the deletion mutants *fzc9*Δ and *pdr802*Δ, with primer details provided in Table S4. The sequence of the Fzc9 homolog gene (CNAG_03059) was retrieved from the *C. neoformans* var. *grubii* serotype A genome database (https://www.broadinstitute.org/fungal-genome-initiative/cryptococcus-neoformans-serotype-genome-project, accessed on 17 February 2025). To generate the *fzc9*Δ mutant, we replaced the genetic locus encompassing a 2927-base pair (bp) open reading frame using homologous recombination with a gene-specific deletion cassette, amplified with the primers Fzc9-UP-F, Fzc9-UP-R, neoF, neoR, Fzc9-Down-F, and Fzc9-Down-R. This deletion cassette was introduced into the wild-type strain via biolistic transformation, as previously described. Positive transformants were verified through polymerase chain reaction (PCR) analysis. Similarly, the sequence for the Pdr802 homolog gene (CNAG_03894) was obtained from the *C. neoformans* var. *grubii* serotype A genome database (https://www.broadinstitute.org/fungal-genome-initiative/cryptococcus-neoformans-serotype-genome-project). To construct the *pdr802*Δ mutant, the genetic locus containing the 2087 bp open reading frame was replaced by homologous recombination with a gene-specific deletion cassette, amplified using the primers Pdr802-UP-F, Pdr802-UP-R, neoF, neoR, Pdr802-Down-F, and Pdr802-Down-R. This deletion cassette was introduced into the WT strain through biolistic transformation, following previously established protocols [24,25,26]. Positive transformants were confirmed via PCR. Fungal cells were cultured overnight in YPD medium with shaking at 150 rpm at 30 °C. After cultivation, the cells intended for proteomic and metabolomic analysis were harvested and washed with PBS. They were then snap-frozen in liquid nitrogen and stored at −80 °C for later analysis.

### 4.2. Proteome Studies

Frozen cells were initially ground in liquid nitrogen to obtain a fine powder. This powder was then subjected to sonication on ice for 3 min in a lysis buffer that included Triton X-100, dithiothreitol, protease inhibitors, and phosphatase inhibitors. Following this, Tris-saturated phenol (pH 8.0) was added, and the mixture was vortexed for 5 min. Centrifugation at 4 °C for 10 min allowed for the separation of phases, after which the upper phenol layer was carefully transferred to a new tube. Ammonium sulfate-saturated methanol was then mixed in, and the sample was incubated at −20 °C for a minimum of 6 h. Upon centrifugation, the protein pellet was washed three times with ice-cold methanol and acetone. The resulting protein was re-dissolved in 8 M urea, with concentration assessed using a BCA kit. To precipitate proteins, trichloroacetic acid was slowly added to achieve a final concentration of 20% (m/v), and the mixture was vortexed before being incubated at 4 °C for 2 h. The precipitated protein was then collected by centrifugation, washed, and dried for 1 min. The dried protein sample was subsequently dissolved in 200 mM TEAB and subjected to ultrasonic dispersion. Trypsin was added at a mass ratio of 1:50 relative to the protein for overnight digestion. The mixture was reduced using 5 mM dithiothreitol at 56 °C for 30 min, followed by alkylation with 11 mM iodoacetamide for 15 min in the dark. Finally, the peptides underwent desalting through a Strata X SPE column. Tryptic peptides were first reconstituted in solvent A before being loaded onto a custom-designed reversed-phase analytical column, which measured 25 cm in length with an inner diameter of 100 μm. The mobile phase consisted of solvent A (0.1% formic acid and 2% acetonitrile in water) and solvent B (0.1% formic acid in acetonitrile). Peptide separation was achieved using a gradient program: from 0 to 14 min, the concentration of solvent B increased from 6% to 24%; between 14 to 16 min, it further rose from 24% to 35%; then from 16 to 18 min, it surged to 90%; and finally, it was maintained at 90% B from 18 to 20 min. This was conducted at a consistent flow rate of 500 nL/min on an Easy-nLC1000 UHPLC system (Bruker Daltonics, Billerica, MA, USA). The eluted peptides were subsequently analyzed via a capillary source connected to a timsTOF Pro mass spectrometer, operating under an electrospray voltage of 1.75 kV. The TOF mass detector was utilized to detect both precursor ions and their fragments. The timsTOF Pro was set to operate in data-independent parallel accumulation serial fragmentation (dia-PASEF) mode. The full MS scan range was established between 300 and 1500 *m/z*, capturing 20 PASEF-MS/MS scans per cycle. The range for MS/MS scans was defined from 400 to 850 *m*/*z*, with an isolation window of 7 *m*/*z*. For data processing, the DIA-NN search engine (version 1.8) was employed. Tandem mass spectra were matched against the *Cryptococcus neoformans* var. *grubii* serotype A strain H99 235443 PR 20240409.fasta database, which included 7429 entries, augmented with a reverse decoy database. Trypsin/P was specified as the cleavage enzyme, allowing for a maximum of one missed cleavage. Fixed modifications accounted for N-terminal methionine excision and carbamidomethylation of cysteine residues. Importantly, the false discovery rate was stringently controlled to remain below 1%. The Gene Ontology (GO) annotation process began with the utilization of eggnog-mapper software v2.1.12 to retrieve GO IDs for the identified proteins, leveraging the information available in the EggNOG database. Following this, we conducted a functional classification analysis that categorized proteins into three distinct areas: cellular components, molecular functions, and biological processes. To annotate protein pathways, we relied on the KEGG pathway database, employing BLAST comparisons (BLAST+ 2.13.0) (specifically blastp, with an e-value threshold of ≤1 × 10^−4^) to identify proteins. For each sequence, the annotation was derived from the highest scoring comparison result. In addition, PSORTb software v3.0.2 was employed to predict the subcellular localization of the four classes of proteins identified in prokaryotes. To assess the significance of functional enrichment among differentially expressed proteins, we applied Fisher’s exact test, using the identified proteins as the background. Functional terms with a fold enrichment greater than 1.5 and a *p*-value less than 0.05 were deemed statistically significant.

### 4.3. Metabolomics Studies

The process of sample preparation for metabolomics studies commenced with the careful thawing of cell samples previously stored at −80 °C, ensuring that they are kept on ice throughout. The samples were then homogenized for a duration of 20 s using a grinder, after which they were combined with 400 μL of a methanol-water solution (4:1, v/v) that included an internal standard. The resulting mixture was vortexed for 3 min to ensure thorough mixing. Subsequently, the samples underwent three freeze-thaw cycles, alternating between liquid nitrogen and dry ice, followed by additional vortexing to enhance the extraction process. The samples were then centrifuged at 12,000 rpm for 10 min at 4 °C, allowing for the collection of 300 μL of the supernatant. This supernatant was stored at −20 °C for 30 min prior to a second centrifugation step. A 200 μL aliquot of the supernatant was then prepared for LC-MS analysis. For the analysis, all samples were subjected to two separate LC-MS methodologies. One aliquot was analyzed under positive ionization conditions using a T3 column (Waters ACQUITY Premier HSS T3 Column, 1.8 µm, 2.1 mm × 100 mm). The mobile phase consisted of 0.1% formic acid in water (solvent A) and 0.1% formic acid in acetonitrile (solvent B), following a specific gradient schedule: initiating from 5% B, increasing to 20% B over 2 min, rising to 60% B in the following 3 min, escalating to 99% B within 1 min, maintaining this concentration for 1.5 min, and finally reverting to 5% B within 0.1 min, followed by a 2.4-min hold. The analytical setup included a column temperature set at 40 °C, a flow rate of 0.4 mL/min, and an injection volume of 4 μL. A second aliquot was analyzed under negative ionization conditions, utilizing the same elution gradient as that for the positive mode. Data acquisition occurs in information-dependent acquisition mode with Analyst TF 1.7.1 Software (Sciex, Concord, ON, Canada). The parameters for the ion source were configured as follows: ion source gas 1 at 50 psi; ion source gas 2 at 50 psi; curtain gas at 25 psi; temperature set to 550 °C; declustering potential at 60 V for the positive mode and −60 V for the negative mode; and ion spray voltage floating at 5000 V for positive and −4000 V for negative ionization conditions. The Time-of-Flight (TOF) MS scan parameters were defined with a mass range of 50–1000 Da, an accumulation time of 200 ms, and dynamic background subtraction activated. In product ion scans, the parameters included a mass range of 25–1000 Da, an accumulation time of 40 ms, collision energies of 30 V for the positive mode and −30 V for the negative mode, a collision energy spread of 15, a resolution set to UNIT, charge state limited to 1, intensity set at 100 counts per second, exclusion of isotopes within 4 Da, a mass tolerance of 50 ppm, and a maximum of 18 candidate ions monitored per cycle. Differential metabolites were identified using Variable Importance in Projection (VIP) values (VIP > 1) alongside *p*-values (*p* < 0.05) through Orthogonal Partial Least Squares Discriminant Analysis. Furthermore, KEGG annotation and enrichment analysis were performed to pinpoint significantly enriched pathways using hypergeometric testing. To investigate potential relationships between differentially expressed proteins and differential metabolites within particular pathways, separate KEGG pathway annotation analyses were conducted for both categories. Pathways that encompassed both differentially expressed proteins and metabolites were subsequently identified.

### 4.4. Assessment of Intracellular ATP Levels

For the assessment of intracellular ATP levels, cells were cultured overnight in YPD medium at 30 °C with shaking at 150 rpm. ATP concentrations were quantified using the BacTiter-Glo Microbial Cell Viability Assay Kit (Promega, Madison, WI, USA), following the instructions provided in the Kit, and were measured with a microplate reader (TECAN Infinite E Plex, Männedorf, Switzerland). The experiments were conducted with three biological replicates.

## Data Availability

The mass spectrometry proteomics data can be accessed through the ProteomeXchange Consortium, specifically under dataset PXD060124 in the PRIDE repository. In addition, the metabolomics data has been archived in the EMBL-EBI MetaboLights database, where it is designated as MTBLS12237.

## Supplementary Materials

The supporting information can be downloaded at: https://www.mdpi.com/article/10.3390/ijms26051824/s1.
