# Supplementary material for: Transcription Factors Fzc9 and Pdr802 Regulate ATP Levels and Metabolism in Cryptococcus neoformans
Source: Int J Mol Sci. 2025 Feb 20;26(5):1824. doi: 10.3390/ijms26051824 (PMC11899616; doi:10.3390/ijms26051824)
Supplement: Supplementary file 1 [file ijms-26-01824-s001.zip › Supplementary tables.docx]

**Table S1** Summary of differentially expressed proteins in the mutant strains *fzc9*Δ and *pdr802*Δ. FC indicates fold change.

| Knockout | Regulated type | FC >1.2 or <0.83 | FC >1.3 or <0.77 | FC >1.5 or <0.67 | FC >2 or <0.5 |
| --- | --- | --- | --- | --- | --- |
| *FZC9* | Up | 334 | 224 | 130 | 39 |
|  | Down | 270 | 174 | 85 | 21 |
| *PDR802* | Up | 360 | 276 | 156 | 55 |
|  | Down | 324 | 207 | 103 | 25 |

**Table S2** Differentially expressed mitochondrial proteins between the WT control and *fzc9*Δ mutant. Proteins with a ratio (*fzc9*Δ mutant / WT control) showing a fold change greater than 1.5 or less than 0.67 and a p-value under 0.05 were considered significantly different.

| Protein | Function | Ratio (WT vs *fzc9*Δ) |
| --- | --- | --- |
| CNAG_02359 | 40S ribosomal protein S25 | 1.94 |
| CNAG_05038 | Uncharacterized protein | 2.04 |
| CNAG_04751 | EF-hand domain-containing protein | 1.85 |
| CNAG_07965 | NAD binding dehydrogenase | 1.6 |
| CNAG_03377 | Uncharacterized protein | 1.54 |
| CNAG_07659 | SH3 domain-containing protein | 2.05 |
| CNAG_02943 | Cytoplasmic protein | 1.53 |
| CNAG_00897 | Glucosidase | 1.63 |
| CNAG_02925 | D-arabinitol 2-dehydrogenase | 1.6 |
| CNAG_07802 | Class III aminotransferase | 2.71 |
| CNAG_04267 | Mitochondrial genome maintenance protein MGM101 | 1.54 |
| CNAG_01181 | Large ribosomal subunit protein eL42 | 1.88 |
| CNAG_00248 | Vacuolar protein-sorting-associated protein 36 | 1.55 |
| CNAG_03240 | alpha-1,2-Mannosidase | 3.07 |
| CCP1 | Cytochrome c peroxidase, mitochondrial | 1.61 |
| CNAG_03747 | Large subunit ribosomal protein L27Ae | 1.51 |
| CNAG_06644 | C-22 sterol desaturase | 0.52 |
| CNAG_05573 | Cytochrome c oxidase assembly protein subunit 17 | 0.59 |
| CNAG_04757 | Mitochondrial protein | 0.47 |
| CNAG_06094 | Uncharacterized protein | 0.33 |
| CNAG_06096 | Tricarboxylate carrier | 0.58 |
| CNAG_05468 | AP endonuclease 1 | 0.66 |
| CNAG_04169 | Holo-[acyl-carrier protein] synthase | 0.66 |
| CNAG_04761 | Ras family protein | 0.59 |
| CNAG_00967 | Endoplasmic reticulum protein | 0.65 |
| CNAG_02288 | Solute carrier family 25 (Mitochondrial citrate transporter), member 1 GN=CNAG_02288 PE=3 SV=1 | 0.44 |
| CNAG_03280 | Dihydrodipicolinate synthetase | 0.61 |
| CNAG_05829 | MIF4G domain-containing protein | 0.62 |
| CNAG_04859 | Rho family protein | 0.6 |
| CNAG_03170 | Deacetylase sirtuin-type domain-containing protein | 0.5 |
| CNAG_03033 | Protein arginine methyltransferase NDUFAF7 | 0.64 |
| CNAG_04088 | Uncharacterized protein | 0.5 |
| CNAG_04031 | rRNA methyltransferase 2, mitochondrial | 0.32 |
| CNAG_07351 | PIN domain-containing protein | 0.62 |
| CNAG_00442 | Cyclin | 0.58 |
| CNAG_00452 | Isovaleryl-CoA dehydrogenase | 0.64 |
| CNAG_00573 | NADH dehydrogenase (Ubiquinone) 1 alpha subcomplex 6 | 0.65 |
| CNAG_02750 | Uncharacterized protein | 0.62 |

**Table S3** Mitochondrial proteins that differ between the WT control and *pdr802*Δ mutant. Significant differences were identified by a (*pdr802*Δ mutant / WT control) ratio with fold changes above 1.5 or below 0.67, and a p-value of less than 0.05.

| Protein | Function | Ratio (WT vs *pdr802*Δ) |
| --- | --- | --- |
| CNAG_07659 | SH3 domain-containing protein | 1.68 |
| CNAG_01261 | Myosin I binding protein | 1.52 |
| CNAG_03127 | Small subunit ribosomal protein S23 | 2.75 |
| CNAG_03002 | Uncharacterized protein | 1.87 |
| CNAG_07965 | NAD binding dehydrogenase | 1.65 |
| CNAG_04694 | Periodic tryptophan protein 1 | 2.24 |
| CNAG_06007 | U3 small nucleolar RNA-associated protein 23 | 1.51 |
| CNAG_02943 | Cytoplasmic protein | 1.8 |
| CNAG_07426 | Proteasome maturation protein | 1.69 |
| CNAG_00897 | Glucosidase | 1.58 |
| CCP1 | Cytochrome c peroxidase, mitochondrial | 1.59 |
| CNAG_04011 | Large ribosomal subunit protein eL42 | 1.67 |
| CNAG_05038 | Uncharacterized protein | 1.61 |
| CNAG_00647 | Uncharacterized protein | 1.67 |
| CNAG_02925 | D-arabinitol 2-dehydrogenase | 1.55 |
| CNAG_00149 | NADH dehydrogenase (Ubiquinone) 1 alpha subcomplex 4 | 2.51 |
| CNAG_03747 | Large subunit ribosomal protein L27Ae | 1.7 |
| CNAG_03667 | Uncharacterized protein | 2.08 |
| CNAG_03240 | alpha-1,2-Mannosidase | 3.23 |
| CNAG_07802 | Class III aminotransferase | 2.87 |
| CNAG_05859 | Uncharacterized protein | 0.48 |
| CNAG_01323 | Complex III subunit 7 | 0.57 |
| CNAG_02288 | Solute carrier family 25 (Mitochondrial citrate transporter), member 1 | 0.49 |
| CNAG_06644 | C-22 sterol desaturase | 0.46 |
| CNAG_06184 | SWI5-dependent HO expression protein 3 | 0.66 |
| CNAG_01534 | Gram-positive cocci surface proteins LPxTG domain-containing protein | 0.27 |
| CNAG_06226 | NADH dehydrogenase (Ubiquinone) 1 alpha subcomplex 5 | 0.51 |
| CNAG_01752 | Solute carrier family 25 (Mitochondrial 2-oxodicarboxylate transporter), member 21 | 0.62 |
| CNAG_06094 | Uncharacterized protein | 0.37 |
| CNAG_04757 | Mitochondrial protein | 0.39 |
| CNAG_05573 | Cytochrome c oxidase assembly protein subunit 17 | 0.64 |
| CNAG_06358 | Glutamyl-tRNA(Gln) amidotransferase subunit F, mitochondrial | 0.65 |
| CNAG_05829 | MIF4G domain-containing protein | 0.65 |
| CNAG_00894 | F-type H+-transporting ATPase subunit epsilon | 0.59 |
| CNAG_00453 | Mitochondrial protein | 0.65 |
| CNAG_00516 | Peroxin-7 | 0.59 |
| CNAG_02460 | coproporphyrinogen oxidase | 0.63 |
| CNAG_06782 | Alpha 1,2-mannosyltransferase | 0.66 |
| CNAG_04088 | Uncharacterized protein | 0.51 |
| CNAG_02750 | Uncharacterized protein | 0.61 |
| CNAG_04031 | rRNA methyltransferase 2, mitochondrial | 0.5 |
| CNAG_07356 | Succinate dehydrogenase, cytochrome b556 subunit | 0.62 |
| CNAG_00442 | Cyclin | 0.57 |
| CNAG_00452 | Isovaleryl-CoA dehydrogenase | 0.55 |
| CNAG_00573 | NADH dehydrogenase (Ubiquinone) 1 alpha subcomplex 6 | 0.56 |
| CNAG_00067 | Protein translocase SEC61 complex gamma subunit | 0.63 |
| CNAG_07351 | PIN domain-containing protein | 0.53 |

**Table S4** Primer employed for the knockout of *fzc9*Δ and *pdr802*Δ mutants within the WT strain. F: forward primer; R: reverse primer.

| **Primers for** ***fzc9*Δ mutant in the WT strain** | | |
| --- | --- | --- |
| **Name** | **Sequence 5’-3’** | **Reference** |
| Fzc9-UP-F | acagagtctttcttgccctcaagt | This Study |
| Fzc9-UP-R | tgagtcgtattacaattcactggccgtcgttttacgcggcttgttgaaaatccctttg | This Study |
| neoF | gccggtgttaataataataatgaatctatgatcgggtaaaacgacggccagtgaattgtaatacg | This Study |
| neoR | gaggaaaaaatgataaggtcaacttatactggtatccaggaaacagctatgaccatgattacgc | This Study |
| Fzc9-Down-F | atggtcatagctgtttcctgaaacatgttcatgccttccaattccc | This Study |
| Fzc9-Down-R | cgcatctgcctgataaatgggtt | This Study |
| **Primers for *pdr802*Δ mutant in the WT strain** | | |
| **Name** | **Sequence 5’-3’** | **Reference** |
| Pdr802-UP-F | atgacattacagcagcggc | This Study |
| Pdr802-UP-R | tgagtcgtattacaattcactggccgtcgttttacccgtgttcagatcgatctttttcgg | This Study |
| neoF | gccggtgttaataataataatgaatctatgatcgggtaaaacgacggccagtgaattgtaatacg | This Study |
| neoR | gaggaaaaaatgataaggtcaacttatactggtatccaggaaacagctatgaccatgattacgc | This Study |
| Pdr802-Down-F | atggtcatagctgtttcctggaaaggagggcaatatgggacc | This Study |
| Pdr802-Down-R | ctgatagacgggttcttggctga | This Study |
